# Milk mineral composition is strongly associated with the human milk microbiome

**DOI:** 10.3389/fnut.2025.1550292

**Published:** 2025-05-21

**Authors:** Lilian Lopez Leyva, Emmanuel Gonzalez, Corinne F. Maurice, Kristine G. Koski

**Affiliations:** ^1^School of Human Nutrition, McGill University, Montreal, QC, Canada; ^2^Canadian Centre for Computational Genomics (C3G), Montreal, QC, Canada; ^3^McGill Interdisciplinary Initiative in Infection and Immunity (MI4), McGill University, Montreal, QC, Canada; ^4^Microbiology and Immunology Department, McGill University, Montreal, QC, Canada; ^5^McGill Centre for Microbiome Research, McGill University, Montreal, QC, Canada

**Keywords:** milk minerals, maternal diet, milk microbiome, lactation, 16S rRNA sequencing, *Mam-*Mayan community

## Abstract

**Introduction:**

Associations between maternal mineral intake, human milk mineral concentrations, and their interactions with the milk microbiota remain understudied, especially in low- and middle-income countries. To understand potential interactions and gain insight into milk composition dynamics, we explored associations of milk mineral concentrations with maternal mineral intakes and the human milk microbiome in an indigenous Guatemalan community.

**Methods:**

In this cross-sectional study, milk samples were collected from 77 Mam-Mayan mothers and classified into early and established lactation. Concentrations of 9 milk minerals were analyzed, and maternal dietary intake was obtained from two 24-h recalls. Microbiome diversity was assessed by 16S rRNA gene sequencing (V5–V6 region). DESeq2 was used for differential abundance analysis. PCA and Spearman’s rank correlation explored relationships among milk minerals, maternal mineral intake, and differentially abundant microbial taxa; results with FDR-adjusted *p*-values < 0.1 were retained.

**Results:**

Our multifactorial analysis revealed strong associations between milk minerals and the milk microbiome and weak associations with maternal intake. Several maternal intakes (Ca, Se, K, Fe, Mn) and milk mineral concentrations (Ca, Se, K, Mg, Na) were below reference values. In early lactation, milk Fe, Mn, Se, and Cu correlated with differentially abundant taxa, while in established lactation, Fe, Mn, Se, Ca, and Na were correlated. Fe and Mn accounted for 64% of bacterial associations in early lactation and 75% in established lactation. These minerals were correlated with Pseudomonadota (early), Actinomycetota (established), and Bacillota (both), but all species were unique to each stage.

**Conclusion:**

Our findings reveal a complex interplay between milk minerals and the microbiome. Iron, manganese, and selenium were consistently associated with milk bacteria across lactation stages. These correlations may reflect microbial responses to mineral availability. Further longitudinal studies with larger samples are needed to clarify how this interaction influences mineral bioavailability and infant growth.

## Introduction

Human milk is widely recognized as a complete and optimal source of nutrients for infants (1); however, these assumptions are being challenged, especially for micronutrients, including vitamins and minerals (2). Indeed, recent reports document low concentrations of minerals in human milk that are insufficient to meet the full requirements of the developing infant (3–5).

Prior research had focused on the links between maternal intake of vitamins and fatty acids with milk micronutrient composition (3, 6–8) resulting in the classification of milk nutrients into 2 groups, depending on their association or not with maternal diet (2, 9, 10). Group I nutrients such as thiamin, riboflavin, vitamin B-6, vitamin B-12, choline, retinol, vitamin A, vitamin D, selenium, and iodine were dependent on maternal diet. Supplementing mothers with these nutrients boosted concentrations in human milk and enhanced infant health (4). On the other hand, Group II nutrients such as folate, calcium, iron, copper, and zinc remained largely unchanged in human milk regardless of maternal intake or status (7, 10, 11). In this scenario, maternal supplementation primarily benefited the mother and was not associated with milk composition or infant outcomes (2, 10). The lack of association between maternal mineral intake and milk minerals concentrations might be due to well-regulated homeostatic processes that some minerals like zinc, iron, and copper have, such as active transport mechanisms in the mammary gland (12). However, the available evidence does not report if the lack of association differs according to the adequacy of maternal mineral intake (6, 7, 13). It is therefore possible that the underlying milk mineral homeostatic processes would be distinct between lactating mothers experiencing chronic nutrient deficiencies relative to those with adequate nutrient intakes. The lack of association between maternal mineral intake and milk mineral concentrations supports that other determinants could influence mineral bioavailability in human milk (14).

The collection of microorganisms naturally found in human milk, the milk microbiota, could be interacting with milk minerals and bidirectionally affecting their concentration and bioavailability. Indeed, microorganisms rely on micronutrients for their growth and metabolism (15–17) and several studies have explored these links in the gut (14, 18). For instance, calcium intake has been associated with a higher proportion of *Clostridium* cluster XVIII in the gut microbiota (19), whereas iron supplementation induced a depletion of *Bifidobacterium* and *Lactobacillus* levels in children’s guts (20). However, evidence of such interactions in human milk is scarce. One of the few studies exploring the association between milk minerals and milk microbiota composition reported that calcium was negatively correlated with *Streptococcus, Prevotella*, Actinomycetota, and Bacteroidota; magnesium was positively correlated with *Streptococcus* abundance; and selenium was negatively correlated with *Staphylococcus* (21).

One of the recognized paths of milk microbiome colonization is the entero-mammary route (22–24). Data from animal studies suggest that modulation of the maternal gut microbiota, via diet or probiotics, may influence the milk microbiota composition (25–28). Considering the importance of human milk for infant development, a better understanding of the relationship between milk microbiome composition, milk mineral concentrations, and maternal mineral intake could guide nutritional and microbiome-based interventions in pregnant women and breastfed infants.

Considering that 92% of infants born per minute globally (29) come from developing countries where nutrient deficiencies are common, both for the mother and the infant, it is essential to increase our understanding in these populations. To fill this knowledge gap, we analyzed the associations among milk mineral concentrations, maternal mineral intakes, and human milk microbiome in mothers from eight rural *Mam*-speaking indigenous communities in Guatemala. Our specific objectives were to (1) analyze the association of the maternal mineral intake with milk mineral concentrations, (2) characterize the milk microbiome composition of a group of indigenous mothers from Guatemala, and (3) analyze the associations of milk mineral concentrations and maternal mineral intake with milk microbiome composition.

## Materials and methods

### Study site and participants

This cross-sectional study was part of a collaboration between McGill University and the Center for Studies of Sensory Impairment, Aging and Metabolism (CeSSIAM) in the Republic of Guatemala. Field studies were conducted from June 2012 through January 2013 in eight rural *Mam*-speaking communities of the San Juan Ostuncalco region in Guatemala (30). The inclusion criteria were indigenous lactating women with infants at (1) early stage of lactation (5–46 days) or established stage of lactation (109–184 days) and who (2) had a vaginal delivery and (3) did not report any health issue (30). The exclusion criteria were: (1) mothers with colostrum (milk < 4 days postpartum) and (2) mothers treated with antibiotics during postpartum period. We did not exclude mothers with subclinical mastitis (SCM) in order to maintain sample size; 21% (*n* = 16) of the total sample size had subclinical mastitis, 31.5% (*n* = 12) had SCM in the group of early lactation and 10% (*n* = 4) in the group of established lactation. We did not control for breastfeeding practices, as 77% of mothers exclusively or predominantly breastfed, from which 97% breastfed at early lactation and 56% at established lactation. Maternal age, parity and infant sex were controlled for in the analyses. The participants had low diet diversity (3.4 + 1.3), low food security (38%), and urinary and gastrointestinal infections were rare (5%). This population has been described in detail previously (30). Ethical approval was obtained from the Institutional Review Boards of both institutions and permission was obtained from indigenous community leaders and the local authorities of the Ministry of Health. All mothers provided written informed consent for participation in the study.

### Study design

In this cross-sectional study, milk samples were manually collected from 77 mothers in 2012–2013, with each lactating mother contributing a single milk sample in either early or established lactation. Mothers and their milk were classified into early lactation (5–46 days postpartum) (*n* = 38) and established lactation (109–184 days postpartum) (*n* = 39). These ranges were chosen to be consistent with our previous studies that measured infant growth (5, 31, 32). The power analysis, based on a previous study (33), indicated that a minimum of 20 samples was necessary to reach a power of 0.95 (using a *p*-value correction of 0.001, a minimum fold change of 2, and considering a 12% loss to follow-up). This higher power threshold was selected to offset known limitations in false positive control when using DESeq2 in high-dimensional microbiome datasets and to increase the reliability of observed associations.

### Human milk sample collection

Prior to collection, the nipple and areola of the breast were cleaned with 70% ethyl alcohol. Human milk samples were collected during a 3-h time window in the morning from the breast not recently used for breastfeeding via full manual expression by a trained midwife, who used hand sanitizer before and after collection. This is important since other studies have shown differences in milk microbiome diversity with the use of breast pumps (34–36). Milk was collected into 60 mL plastic vials and immediately placed on ice. Samples were partitioned into 15 mL tubes at the field laboratory, stored at −30°C prior to transfer on dry ice to McGill University where they were stored at −80°C, which is known to preserve the milk microbiome integrity (37), until DNA extraction for microbiome analysis was performed.

### Milk mineral concentrations

Milk concentrations of 9 minerals (Na, Ca, Cu, Fe, Mg, Mn, K, Se, and Zn) were measured as described previously (5, 38). Milk samples were thawed and thoroughly homogenized. Overnight digestion of the samples was completed in plastic DigiTUBEs (SCP Science) through use of trace metal–grade concentrated nitric acid followed by a 3-h reaction with trace metal–grade hydrogen peroxide (30%, Ultrex II, JT Baker), followed by heating at 90°C for 3 h, as previously described (5). Minerals were quantified by inductively coupled mass spectrometry (ICP-MS) using a Varian ICP-820MS (Analytik Jena, Germany) equipped with a Collision Reaction Interface using PlasmaCAL Calibration Standards (SCP Science, Ref #141–110-015). Limits of detection for each of the 9 elements, measured on 8 replicates of the lowest calibration standard were: calcium 1.505 μg/L, copper 0.396 μg/L, iron 1.34 μg/L, magnesium 0.232 μg/L, manganese 0.005 μg/L, potassium 4.887 μg/L, selenium 0.007 μg/L, sodium 1.816 μg/L, and zinc 0.116 μg/L (5, 38). Milk mineral concentrations were compared to the average mineral content in human milk considered by the INCAP (Institute of Nutrition of Central America and Panama) for the Guatemalan population (39).

### Maternal diet assessment

Maternal dietary intake was based on 2 non-consecutive 24 h recalls at either early or established lactation, administered by trained nutritionists as previously described (30). Despite recall bias, underreporting, and difficulty in capturing complex dietary patterns (40), the validity of 2 non-consecutive food frequency questionnaires is similar to the validity of 1–3 days of recall (41). In addition, this approach has been used in previous research projects by CESSIAM (30). Briefly, staff nutritionists at CESSIAM conducted 2 comprehensive quantitative non-consecutive days of 24-h recalls in Spanish or Mam in both early and late lactation. All foods and beverages were recorded and included in the analysis. We acknowledge that the source of dietary minerals (i.e., animal or plants) can affect the bioavailability of the minerals, but the source was not considered in our analysis. This population did not consume any type of supplements, but consumed fortified foods such as “Incaparina,” “Bienestarina,” and “Protemás.” Incaparina is fortified with iron, zinc, and calcium, and also contains added vitamins like vitamin A and B-complex. Bienestarina is fortified with iron, zinc, and calcium, along with added vitamins like vitamin A and folic acid; while Protemás is fortified with iron and zinc, plus other nutrients such as vitamin A and B-complex vitamins. These fortified foods were considered in the evaluation of the maternal mineral intake. The diet data were converted into nutrients for analysis. The nutrients collected were water, energy, lipids, carbohydrates, protein, and 9 minerals (calcium, copper, iron, magnesium, manganese, potassium, selenium, sodium and zinc). The maternal mineral intakes were compared to the daily dietary recommendations by the INCAP for the Guatemalan population (39).

### DNA extraction

DNA extraction was done using 1 mL of milk with the Quick-DNA Fungal/Bacterial Miniprep kit from Zymo Research according to the manufacturer’s protocol. DNA was sent to the McGill Genome Centre for quality control, library preparation, and sequencing. Upon reception of the samples, a Picogreen test was done to measure the DNA concentration. As the concentration of the samples was very low, a speed vac was used to concentrate the DNA before resuspension in 12uL DNAse/RNAse free water. The V5-V6 region was amplified in two steps using the P609F and P699R primers. PCR amplification was performed in triplicate with controls using the NEB Phusion Taq DNA Polymerase with the following cycle conditions: initial denaturation at 98°C for 30 s, followed by 30 cycles of 98°C for 10 s, 58°C for 30 s, 72°C for 30 s, and concluded by a final extension at 72°C for 5 min. The barcoding was done using the Kapa Hifi 2X ready mix with the following conditions: initial denaturation at 95°C for 3 min, followed by 12 cycles of 95°C for 30 s, 55°C for 30 s, 72°C for 30s, and concluded by a final extension at 72°C for 5 min. Following amplicon amplifications, the samples that amplified were pooled and normalized. The normalization was followed by two 1X AMPure clean-up to remove primer-dimers before quantification using qPCR and LabChip. The resulting amplicons were sequenced using an Illumina MiSeq platform at the McGill Genome Centre and assembled from 300 paired-end reads; reagent controls were below the detection limit.

### Microbial community characterization

The sequencing data were processed using the ANCHOR pipeline for microbial analysis (42). Reads were aligned and dereplicated using Mothur (43). Exact sequence variants (ESVs) were selected based on a count threshold of 36 across all samples. Taxonomic annotations were performed using BLASTn against multiple databases, including NCBI 16S RefSeq and NCBI nr/nt, applying criteria of >99% identity and coverage (44, 45). In cases of multiple hits with 100% identity, NCBI 16S rRNA RefSeq was prioritized due to the high standard of curation. All annotations, particularly species calls, should be considered putative even when sharing 100% sequence identity to a single species due to potential errors in databases (42). Given the low biomass nature of these samples, contamination was controlled for at all stages: experimentally with the use of PCR controls and reagent controls (42).

### Microbial community assessment

To visualize the microbial community composition at the phylum level, the relative abundances of exact sequence variants (ESVs) were computed from the normalized data. A custom JavaScript script utilizing the D3.js library (46) was used to create the flower diagram representing cumulative microbial abundance. Alpha and beta diversity analyses were conducted to compare microbial diversity between early and established lactation stages. Alpha diversity was calculated using the Shannon and Simpson indices, while beta diversity was evaluated using Bray–Curtis dissimilarity. The vegan (47), phyloseq (48), and ggplot2 (49) packages in R were used for these calculations and visualizations. Canonical Analysis of Principal Coordinates (CAP) was employed to assess beta diversity and to visualize the separation between the two lactation stages. The significance of group separation was tested using ANOVA-like permutation tests, with *p*-values corrected using the Benjamini-Hochberg procedure.

### Differential abundance analysis

Differential abundance of microbial taxa between early and established lactation stages was assessed using DESeq2 (50). Raw read counts were imported into DESeq2, and regularized log transformation (rlog) was applied as the normalization method to stabilize variance across samples. Differentially abundant exact sequence variants (ESVs) were identified with a significance threshold set at a false discovery rate (FDR) of 0.1. This threshold was chosen due to the multiple testing context of microbiome research and in consideration of the sample size. An FDR < 0.1 allows more leniency while maintaining acceptable control over false positives. For visualization of the results, the phyloseq (48) and ggplot2 (49) R packages were used. Differentially abundant taxa were represented by grouping species by phylum and ordering them according to their log fold change. A dashed red line was used to denote “infinite” log fold changes, indicating that some ESVs were detected in samples from only one group (either early or established lactation) and absent in the other.

### Multifactorial analysis

Principal Component Analysis (PCA) was performed to explore the relationships between milk mineral concentrations, maternal mineral intake, and the differentially abundant microbial taxa. Correlation circles (51) are visual tools derived from PCA to illustrate the relationships between different factors (milk minerals, maternal intake, and microbial taxa) by representing their correlations as angles and their importance to the principal components as their distance from the center and the edge of the circle. The edge of the correlation circle represents a perfect correlation of 1. The closer a variable is to the center of the correlation circle, the weaker its correlation with the principal components being displayed (usually PC1 and PC2), and thus, the less important it is for interpreting these components. On the other hand, the closer a variable is to the edge of the correlation circle, the more of its variance is explained by these components. The angle between the vectors of two variables on the correlation circle reflect their correlation with each other. An acute angle (< 90°) indicates a positive correlation between the two variables. An obtuse angle (> 90°) indicates a negative correlation. A right angle (90°) suggests no correlation between the two variables. Variables that are diametrically opposite on the circle are strongly negatively correlated.

The PCA was conducted using the FactoMineR package (52) in R, with the first five principal components capturing the primary sources of variation in the data. Active variables included the normalized abundances of differentially abundant exact sequence variants (ESVs). Supplementary variables were categorized as either qualitative (anthropologic information) or quantitative (milk nutrients and maternal dietary minerals). The fviz_pca_var function from the factoextra package was used to generate a correlation circle plot, visualizing the relationships and contributions of variables to the principal components.

### Correlation analysis

Spearman’s rank correlation was used to examine the relationships among maternal dietary factors, milk mineral concentrations and differentially abundant bacterial species (ESVs) in early and established lactation samples. The Kendall rank correlation coefficient was calculated using the psych (53) and corrplot (54) R packages. Milk nutrients and dietary minerals were standardized prior to analysis. Bacterial abundance data consisted of ESVs identified as differentially abundant. *p*-values were adjusted for multiple comparisons using the Benjamini-Hochberg method, and results with an adjusted *p*-value (FDR) < 0.1 were retained. ESVs labeled as “Unknown” were excluded from the final heatmap. The correlation matrix was visualized using the corrplot package, where the intensity of color represented the strength of the correlation, and statistical significance was indicated with asterisks.

## Results

### Population characteristics

This cross-sectional study included 77 lactating mothers: 38 (49.4%) were recruited at early stage of lactation (5–46 days) and 39 (50.6%) at established stage (4–6 mo). The average maternal age was 24 yrs.; BMI was 23.5 kg/m^2^ and 70.1% of participants had a normal BMI (18.5–24.9 kg/m^2^) (Supplementary Table S1). Differences in maternal dietary intakes and milk mineral concentrations between early and established lactation are also summarized in Supplementary Table S1.

Higher maternal intakes of water, energy, macronutrients, calcium, selenium, sodium, and lower intakes of zinc were observed during early compared to established lactation. No differences were observed for dietary intake of fiber, copper, iron, magnesium, manganese, and potassium. Milk concentrations of calcium, copper, iron, potassium, selenium, sodium, and zinc were higher during early lactation, whereas magnesium and manganese concentrations in milk did not differ between early and established lactation. Few correlations emerged between maternal mineral intake and milk mineral concentrations that met the criteria for a FDR < 0.1. The few significant correlations were all negative and weak, indicating that maternal mineral intake in our study population did not strongly correlate with milk mineral concentrations (Supplementary Figures S1, S2).

### Comparison of maternal mineral intakes and milk mineral concentrations with INCAP reference standards

**Figure 1:**
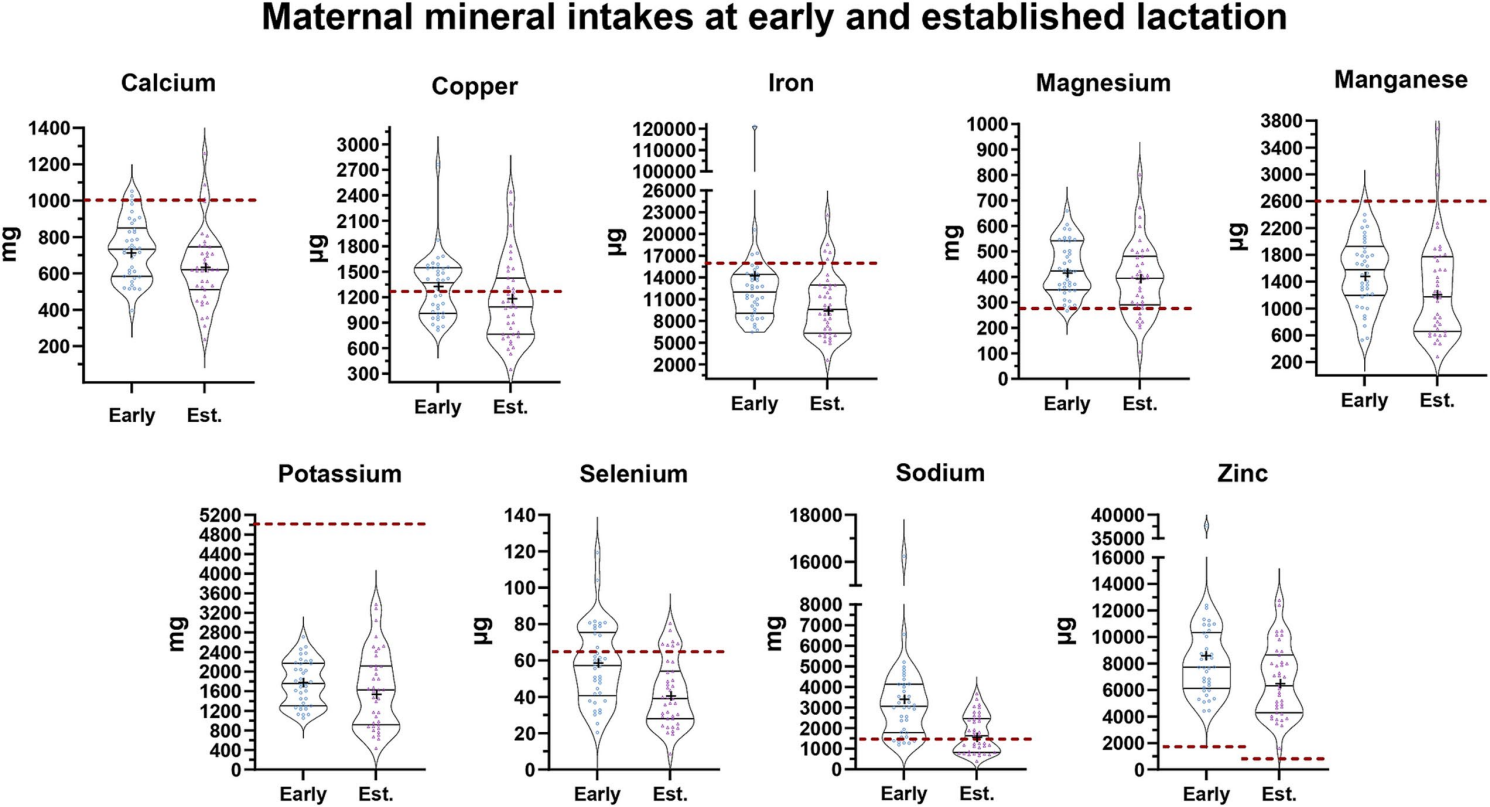
Maternal mineral intake at early and established lactation. Each maternal mineral intake is shown at early (blue) and established (purple) lactation. In each violin plot, the middle line shows the median and all the points represent the spread of the maternal mineral intakes. The length between the top and bottom line represents the interquartile range (IQR, 75 and 25%). The width of the plot represents a more frequent value where it is wider and a less frequent value where it is narrower. The bottom line in the violin plot represents the first quartile and the top line represents the third quartile. The mean is shown as (+). The daily dietary recommendations by the INCAP for the Guatemalan population are shown as a reference in a dashed red line. The reference values for each mineral are the following: [Ca: 1,000 mg, Cu: 1,300 μg, Fe: 15,600 μg, Mg: 275 mg, Mn: 2.6 μg, K: 5,100 mg, Se: 65 μg, Na: 1,500 mg, Zn: 1,700 μg (39)].

A comparison of maternal mineral intake with the daily dietary recommendations of INCAP for the Guatemalan population during early and established lactation is summarized in Figure 1. Considering the interquartile ranges, more than 75% of mothers had intakes of calcium, iron, manganese, and potassium during early and established lactation below INCAP recommendations whereas the majority of mothers exceeded recommendations for magnesium and zinc at both stages of lactation. We also observed that mothers during established lactation had intakes of copper, selenium, and sodium below the INCAP recommendation.

**Figure 2:**
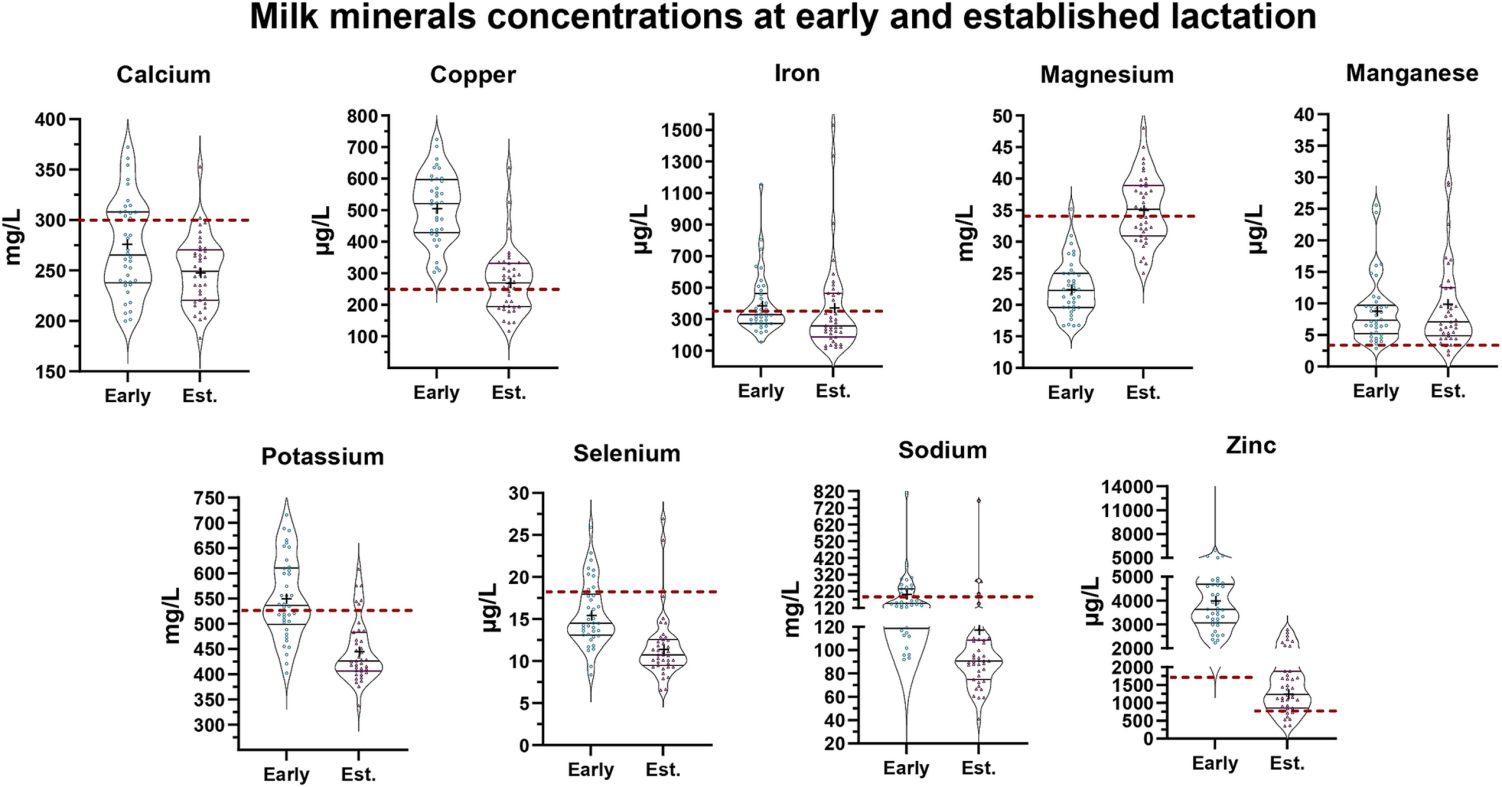
Milk minerals concentrations at early and established lactation. Each milk mineral concentration is shown at early (blue) and established (purple) lactation. In each violin plot, the middle line shows the median and all the points represent the spread of the maternal mineral intakes. The length between the top and bottom line represents the interquartile range (IQR, 75 and 25%). The width of the plot represents a more frequent value where it is wider and a less frequent value where it is narrower. The bottom line in the violin plot represents the first quartile and the top line represents the third quartile. The mean is shown as (+). The average mineral content in human milk considered by the INCAP for the Guatemalan population is shown as a reference in a dashed red line. The reference values for each mineral are the following: Ca: 300 mg/L, Cu: 250 μg/L, Fe: 350 μg/L, Mg: 34 mg/L, Mn: 3 μg/L, K: 525 mg/L, Se: 18 μg/L, Na: 160 mg/L, Zn Early: 1,750 μg/L, Zn Established: 700 μg/L (39).

The comparison of milk mineral concentrations with the recommended INCAP mineral concentrations in human milk also uncovered differences between early and established lactation (Figure 2). Considering the interquartile ranges, we observed that >75% of mothers had milk concentrations below INCAP references during early lactation for calcium, magnesium, and selenium, whereas during established lactation > 75% of mothers had milk concentrations of calcium, selenium, and sodium below the INCAP references. In contrast, milk concentrations of copper, manganese, and zinc at both stages of lactation exceeded the INCAP references (Figure 2).

### Milk microbiome composition during lactation

A total of 2,121 ESVs were assembled and captured 6,539,504 sequence reads across all 77 human milk samples. From those 2,121 ESVs we were able to annotate 1,419 ESVs within 402 genera and 165 bacterial families. The remaining 702 ESVs could not be recognized as 99% similar (in both identity and coverage) to any known taxa and were labeled as “unknown.” However, these ESVs were retained in the statistical analyses to ensure a comprehensive assessment of microbiome composition. Notably, a subset of these ‘unknown’ taxa that were found to be significant in our differential abundance analyses were subsequently identified (100% identity) using BLASTn against a broader reference database. To ensure robust and reliable microbiome comparisons, we used regularized log (RLOG) transformation for normalization. This approach was chosen for its ability to stabilize variance across different abundance levels. To validate the normalization process, we generated abundance vs. variance plots, which confirmed the absence of stochastic effects and ensured appropriate variance stabilization. As contamination is a known challenge in microbiome studies, we included negative (blank) controls during sample processing to monitor potential contaminants.

**Figure 3:**
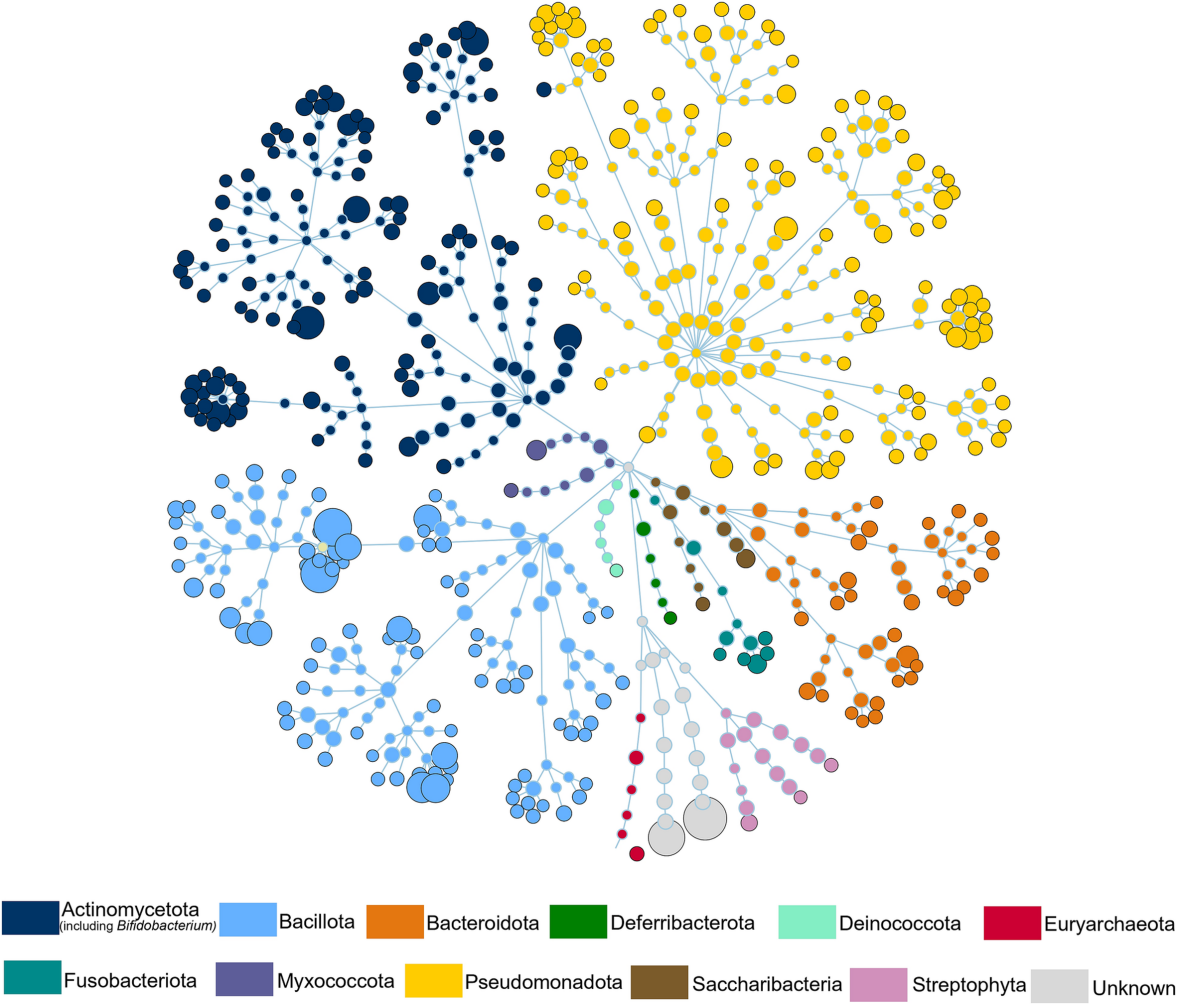
Human milk microbiome composition. The total microbial community colored at phylum level of all the samples.

Of the 1,419 ESVs annotated as putative species, the average BLASTn return identity was 99.9%, including 1,147 perfect hits (100% identity). The most abundant phyla were Pseudomonadota, Bacillota, Actinomycetota, and Bacteroidota (Figure 3). The most abundant ESVs across all samples were *Pseudomonas* spp; *Staphylococcus hominis_2*; and *Streptococcus_MS_12* (which could represent one or more of the species *S. oralis*, *S. mitis*, or *S. tigurinus*) making up 25.91%, 12.37%, and 8.06% of the microbial community, respectively.

**Figure 4:**
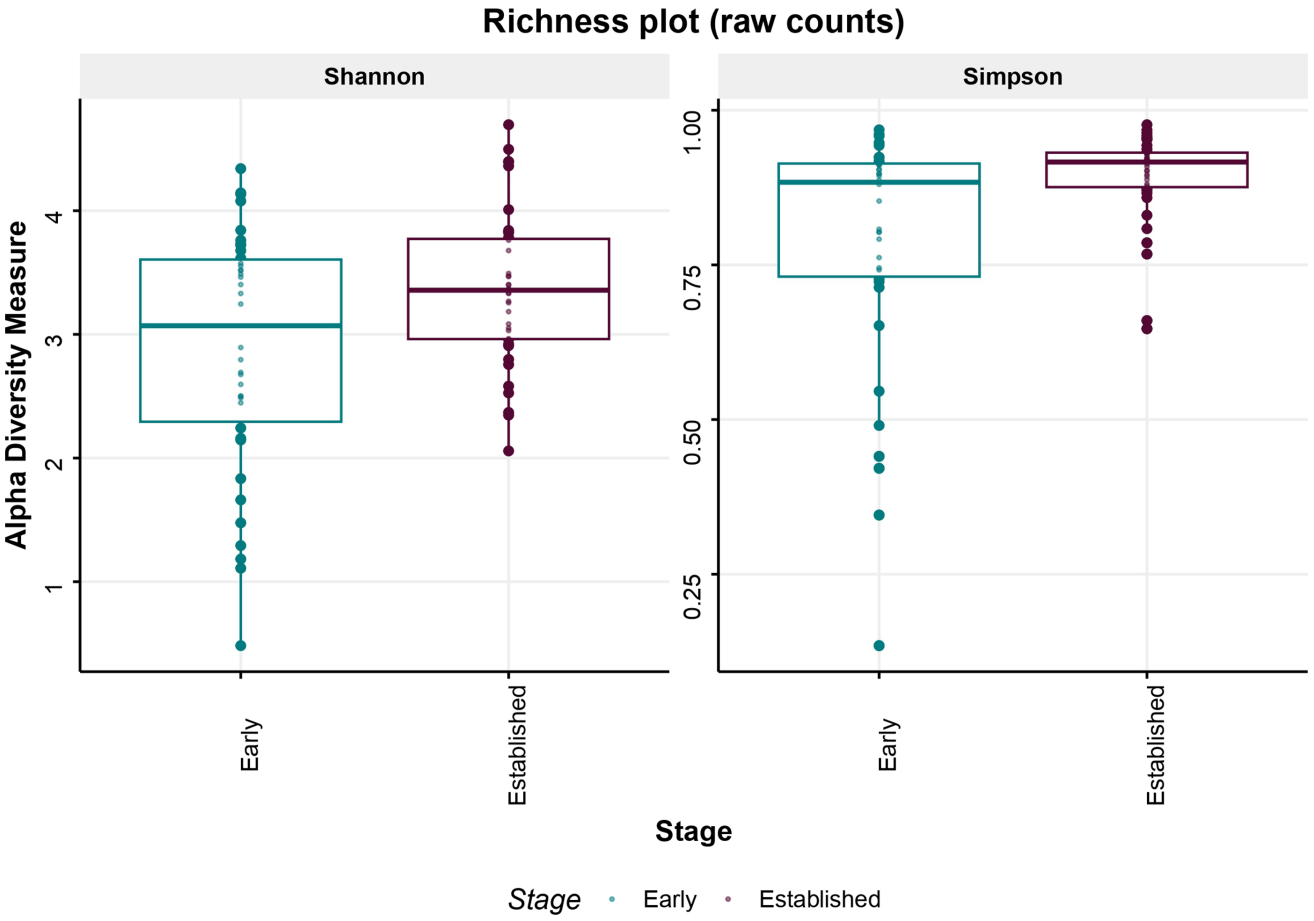
Microbial diversity. Alpha diversity–Microbial diversity indices, Shannon (FDR = 0.01), and Simpson (FDR = 0.008) identified significant differences between early and established lactation.

**Figure 5:**
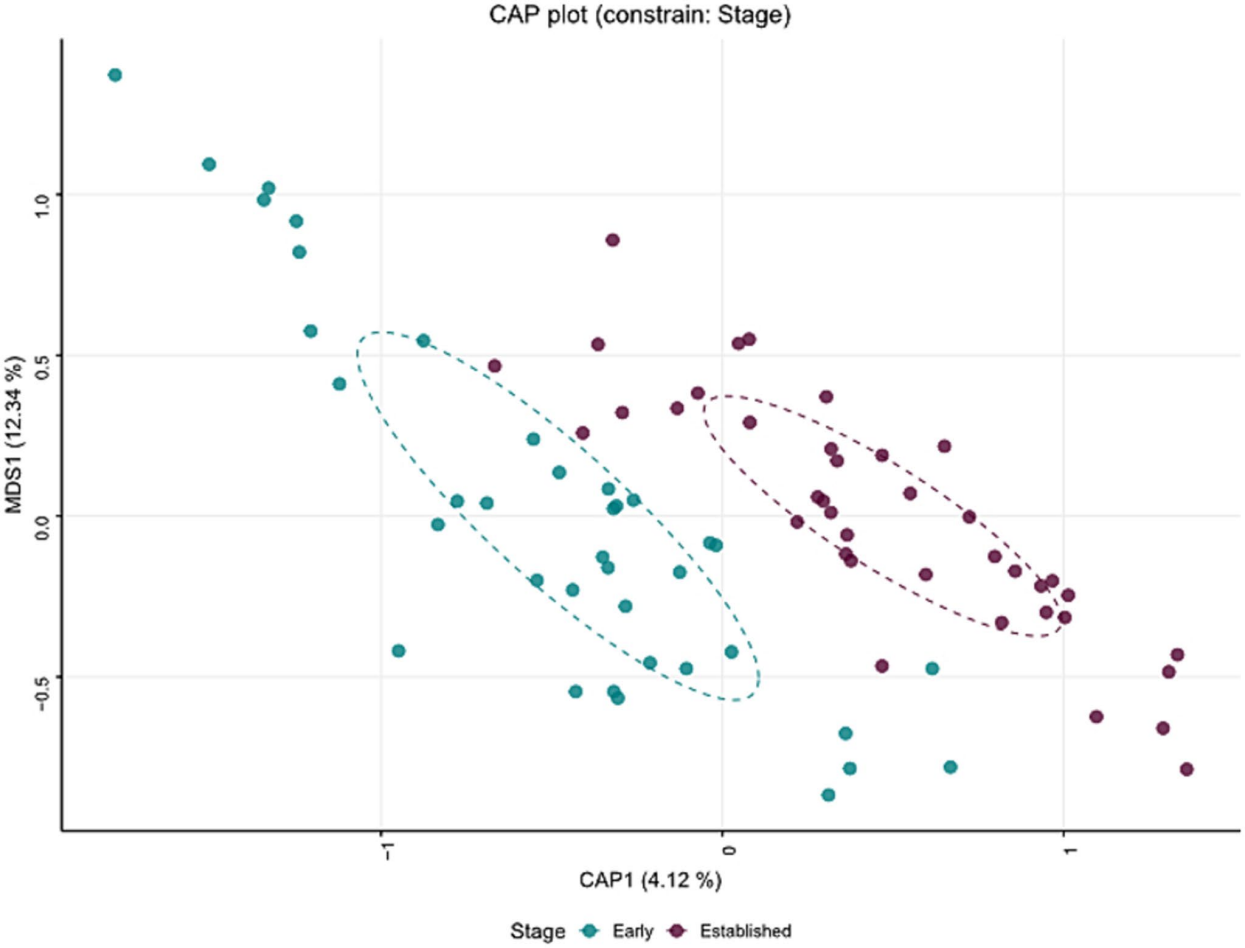
Beta diversity—Main factors were projected onto an unconstrained ordination diagram (NMDS) to reduce data dimensionality, allowing for the visualization of the relationships between samples in a lower-dimensional space (2D) while preserving the rank order of distances. The Canonical Analysis of Principal Coordinates (CAP) ordination segregated both lactation stages (*p* < 0.001).

The estimation of within (alpha) and between (beta) sample diversity in these milk samples is shown in Figures 4, 5, respectively. Microbial diversity indices, such as Shannon (FDR = 0.01), and Simpson (FDR = 0.008) identified significant differences between early and established lactation (Figure 4); the indices Observed and Chao1 were not significant. Beta-diversity analyses segregated both lactation stages, along the Canonical Correspondence Analysis (CCA) (*p* = 1.0E-04), Redundancy Analysis (RDA) (*p* = 2.0E-04) and Canonical Analysis of Principal Coordinates (CAP) (*p* = 1.0E-04) ordinations (Figure 5; Supplementary Figure S3).

### Microbial differential abundance analyses between early and established lactation

**Figure 6:**
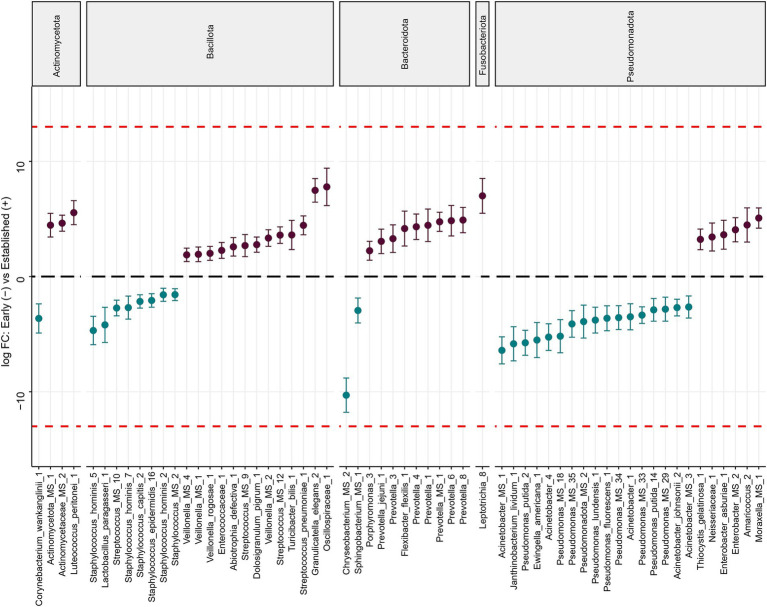
Differentially abundant bacteria associated with the lactation stage. Significantly different ESVs between groups were estimated using DESeq (FDR < 0.1). Species are grouped by phylum and ordered by logFC in each group. The dashed red line indicates “infinite” log fold change, where an ESV had detectable counts in samples from only a single group. Differentially abundant ESVs between the early (*n* = 38) (blue) and established (*n* = 39) (purple) groups. 120 ESVs were differentially abundant, of which 50 were more abundant at early lactation and 70 at established lactation. ESVs labeled as “Unknown” were excluded from the final figure.

Differential abundance analysis (DA) using DESeq2 identified taxa which were significantly different in relative abundance between early and established lactation. In total, 120 ESVs were identified as significantly differentially abundant, including 50 which were in higher relative abundance in early lactation and the remaining 70 higher in established lactation (Figure 6). These could be annotated at various taxonomic levels, spanning 37 species, 26 genera, 26 families, as well as 61 which could not be determined (labeled as “Unknown”). Bacteria from the Bacillota phylum, a common abundant gut bacterial phylum, were similarly present in early (8 ESVs) and established lactation (13 ESVs). Early lactation was dominated by members of the Pseudomonadota phylum (17 ESVs in early lactation vs. 6 ESVs in established lactation). In contrast, taxa from the Actinomycetota (3 ESVs vs. 1 ESVs), Bacteroidota (9 ESVs vs. 2 ESVs), and Fusobacteriota (1 ESVs vs. 0 ESVs) phyla dominated at established lactation.

The ESVs identified as having significantly higher relative abundance in early lactation and that had the highest fold change (FC) were *Acinetobacter* spp. (FC = −6.1), and *Chryseobacterium* spp. (FC = −10.3), while at established lactation were *Granulicatella elegans* (FC = 7.5) and *Leptotrichia_8* (FC = 7). The other ESVs shown in the figure were also significant but had smaller fold changes (Figure 6).

### Multifactorial associations among maternal diet, milk mineral composition, and the milk microbiome

**Figure 7:**
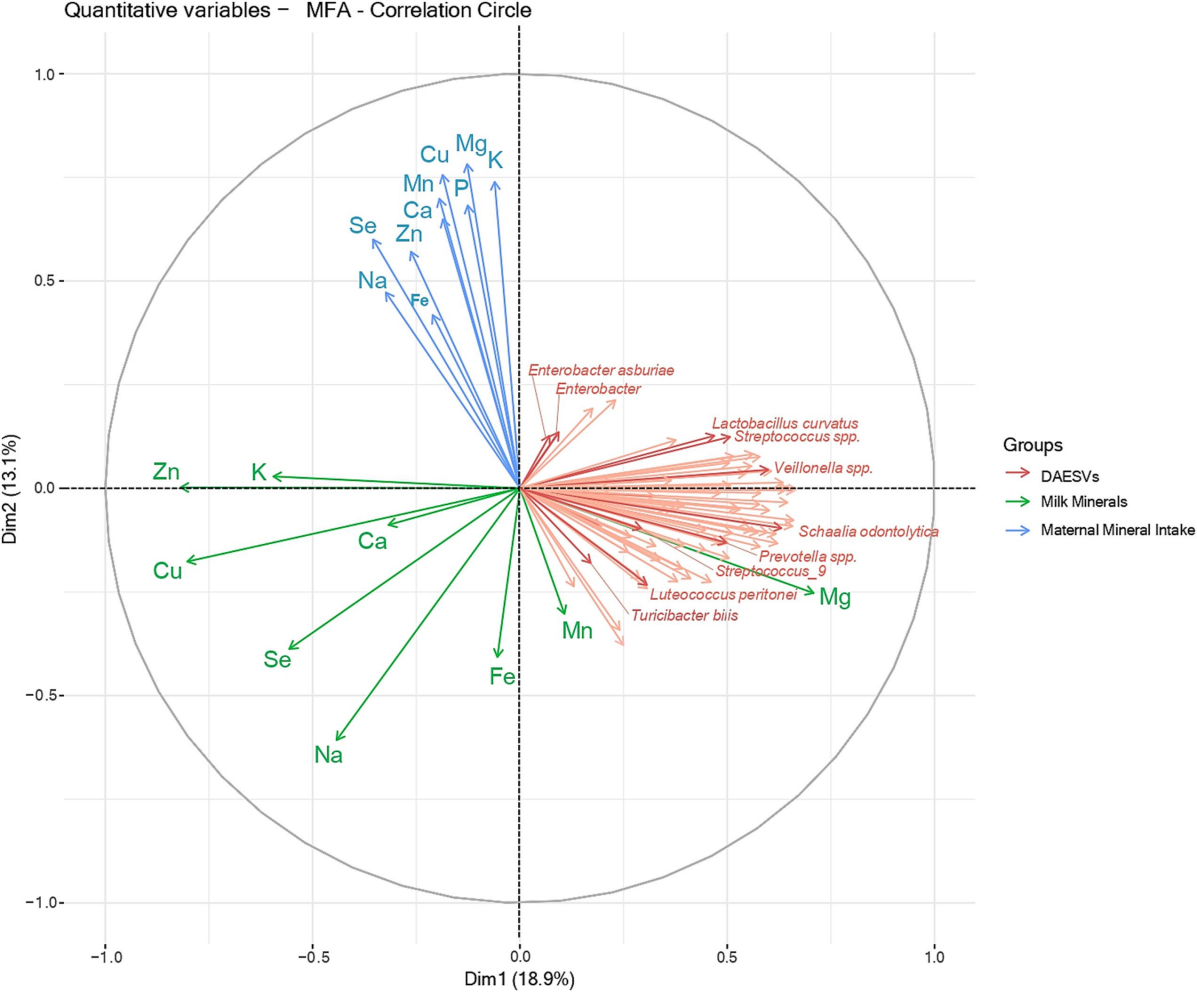
Multifactorial analysis between differential abundant ESVs, milk minerals and maternal mineral intake. The correlation circle represents the multifactorial analysis between the differential abundant ESVs (pink), milk minerals concentrations (green) and maternal mineral intake (blue). The interpretation of a correlation circle considers 3 elements: distance from the center, proximity to the edge of the circle and the angles between the variable vectors. Therefore, the closer a variable is to the center of the correlation circle, the weaker its correlation with the principal components being displayed. The closer a variable is to the edge of the correlation circle, the better it is represented by the principal components being displayed. The angle between the vectors (from the origin to the variable points) of two variables on the correlation circle reflects their correlation with each other. An angle of 90° shows no correlation between variables, an angle > 90° shows a negative correlation and an angle < 90° shows a negative correlation. Thus, no direct associations were observed between milk mineral concentrations (green) and maternal minerals intake (blue) (90° angle). However, most milk mineral concentrations (green) were negatively associated with milk bacteria (pink), as vectors are pointing in opposite directions (180° angle). Milk manganese and milk magnesium are in the same direction and close to the ESVs vectors (angle < 90°), meaning these milk minerals are positively correlated with milk bacteria. Milk bacteria is represented by the ESVs that were differentially abundant between early and established lactation (DA, Differentially abundant; ESV, Exact Sequence variant).

The multifactorial analysis between milk mineral concentrations, maternal mineral intake, and milk microbiome composition shows weak associations between maternal mineral intake and milk mineral concentrations, whereas milk mineral concentrations were negatively associated with milk bacterial diversity (Figure 7). Milk manganese and milk magnesium are in the same direction and close to the ESVs vectors, meaning that these milk minerals are positively correlated with the milk microbiome.

**Figure 8:**
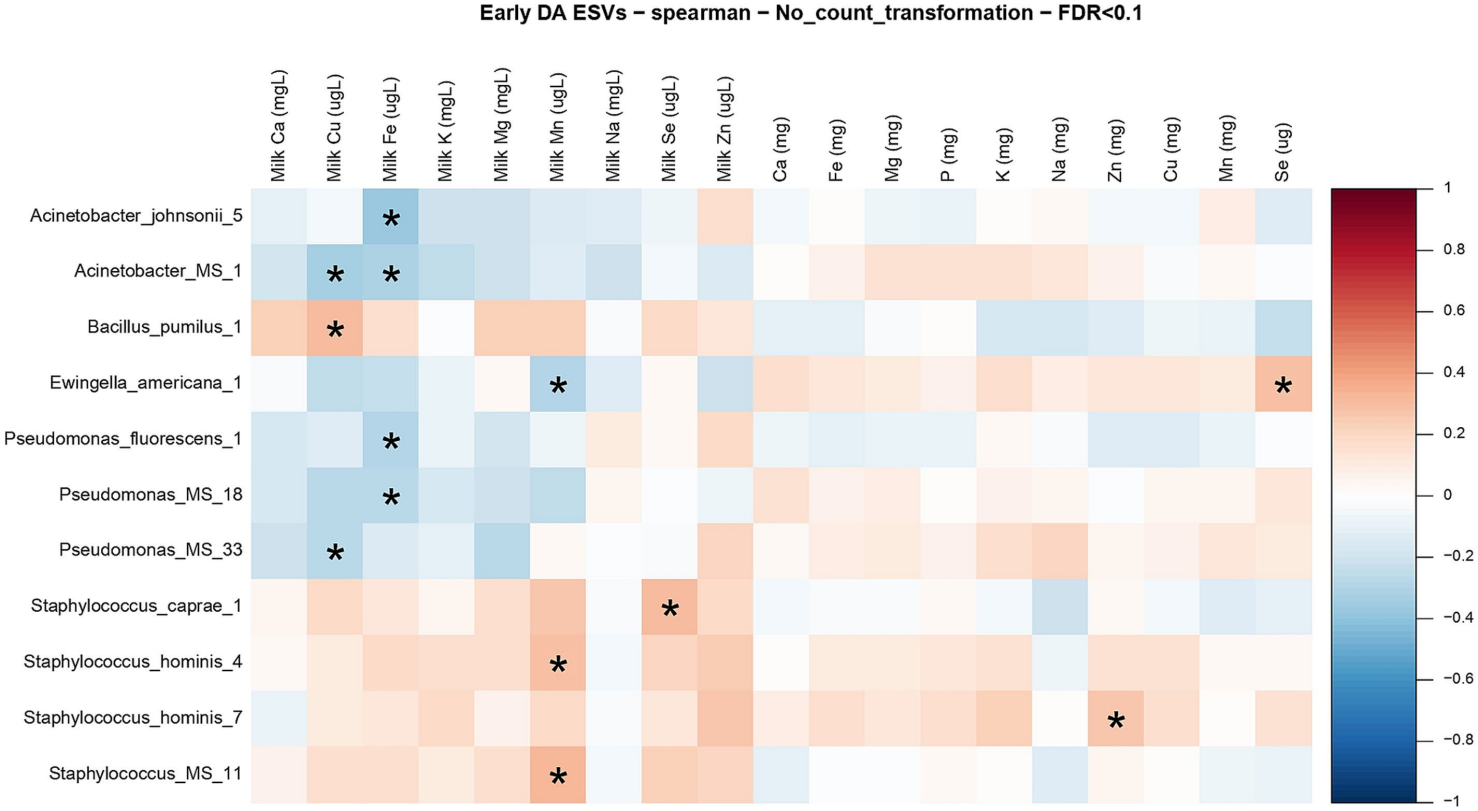
Correlation matrix between differentially abundant ESVs, milk mineral concentrations, and maternal mineral intake at early lactation. Heatmap representing the bivariate Spearman correlation matrix between differentially abundant ESVs, milk mineral concentrations, and maternal mineral intake at early lactation. In the heatmap, the y-axis represents the differentially abundant ESV’s. In the x-axis the minerals preceded by “milk” and with the units (mg/L or μg/L) represent the milk mineral concentrations and the minerals with the units in (mg) represent the maternal mineral intake. The red squares represent positive correlations, and the blue squares represent negative correlations. The intensity of the colors represents the degree of association between the ESVs and the milk mineral concentrations as measured by Spearman’s correlations. The stars represent significant correlations (FDR < 0.1).

**Figure 9:**
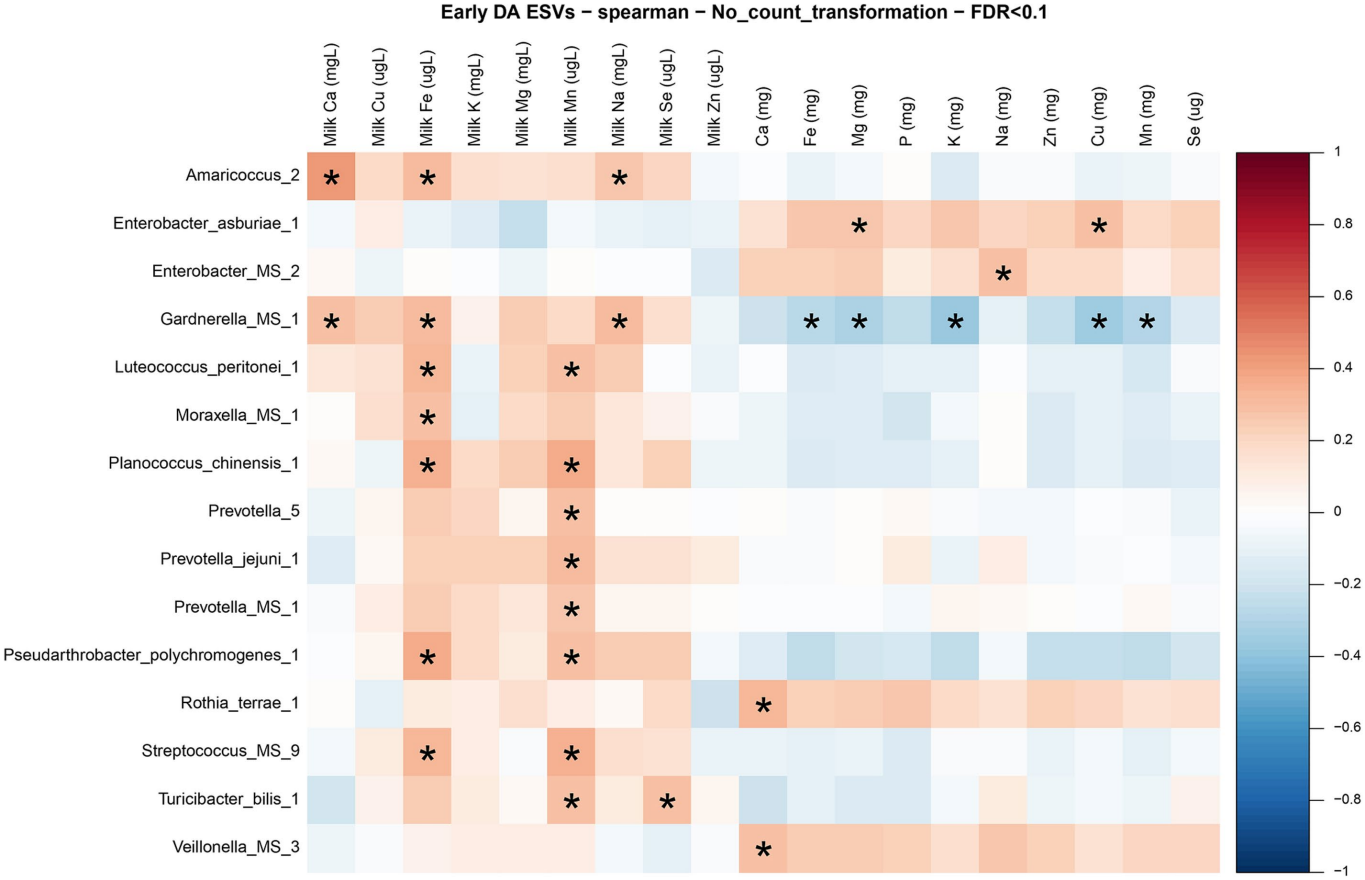
Correlation matrix between differentially abundant ESVs, milk mineral concentrations, and maternal mineral intake at established lactation. Heatmap representing the bivariate Spearman correlation matrix between differentially abundant ESVs, milk mineral concentrations, and maternal mineral intake at established lactation. In the heatmap, the y-axis represents the differentially abundant ESV’s. In the x-axis the minerals preceded by “milk” and with the units (mg/L or μg/L) represent the milk mineral concentrations and the minerals with the units in (mg) represent the maternal mineral intake. The red squares represent positive correlations, and the blue squares represent negative correlations. The intensity of the colors represents the degree of association between the ESVs and the milk mineral concentrations as measured by Spearman’s correlations. The stars represent significant correlations (FDR < 0.1).

Given the mineral and microbial differences between early and established lactation (Figures 5, 6), we proceeded to explore associations between maternal diet, milk mineral composition, and milk microbial diversity within each stage of lactation in Figures 8, 9. The heatmap in Figure 8 shows the bivariate correlations between the differentially abundant ESVs with milk minerals and maternal mineral intake at early lactation. The milk minerals with more correlations in descending order were iron (4), manganese (3), copper (3), and selenium (1). Iron and manganese accounted for 64% of all the associations. Iron in the milk was negatively correlated with *Acinetobacter johnsonii, Acinetobacter* spp.*, Pseudomonas fluorescens,* and *Pseudomonas* spp. Milk manganese was negatively correlated with *Ewingella americana* and positively correlated with *Staphylococcus hominis* and *Staphylococcus* spp. Milk copper was also negatively correlated with *Acinetobacter* spp. and *Pseudomonas* spp., while it was positively correlated with *Bacillus pumilus.* Finally, milk selenium was positively correlated with *Staphylococcus caprae.* In relation to maternal mineral intake, only dietary selenium was positively correlated with *E. americana,* and dietary zinc was positively correlated with *S. hominis.*

The heatmap in Figure 9 shows the correlations between the differentially abundant ESVs with milk minerals and maternal mineral intake at established lactation. The milk minerals with more correlations in descending order were manganese (8), iron (7), calcium (2), sodium (2), and selenium (1). All these correlations were positive. Iron and manganese accounted for 75% of all the associations. Some taxa were correlated with more than one mineral. For instance, *Amaricoccus* spp. and *Garnderella* spp. were correlated with milk calcium, iron, and sodium; *Luteococcus peritonei, Planococcus chinesis, Pseudarthrobacter polychromogenes,* and *Streptococcus* spp. were correlated with milk iron and manganese. Finally, *Turicibacter bilis* was correlated with milk manganese and selenium. Regarding the maternal mineral intakes, dietary calcium was positively correlated with *Rothia terrae* and *Veillonella* spp., dietary sodium was positively correlated with *Enterobacter* spp., dietary magnesium and copper were positively correlated with *Enterobacter asburiae* and dietary iron, magnesium, potassium, copper, and manganese were negatively correlated with *Gardnerella* spp. Moreover, *Bifidobacteria* and *Lactobacillus* were not significantly associated with any milk mineral, nor maternal mineral intake at both lactation stages.

## Discussion

To the best of our knowledge the current study is the first to analyze collectively associations between concentrations of milk minerals and maternal mineral intakes with the human milk microbiome in an Indigenous population of Guatemala. The primer selection (V5-V6) used in this study allowed us to capture a wide range of bacteria, including *Bifidobacteria,* which are typically not captured when using other primers and regions such as V3-V4 (55). Our population also provided a unique opportunity to analyze the milk microbiome, as many of the factors known to affect its composition are homogeneous in the *Mam*-Mayan mothers, including exclusive or partial breastfeeding for >6 months (56), the absence of therapeutic use of antibiotics, and manual milk extraction rather than the use of a breast pump (34, 35, 57).

Our multifactorial analysis among maternal diet, milk mineral concentrations, and the milk microbiome identified strong associations of milk mineral concentrations with the milk microbiome. All associations reported are exploratory in nature and do not imply causation. Early lactation was dominated by Pseudomonadota taxa, whereas Actinomycetota, Bacteriodota, and Fusobacteriota dominated at established lactation. The Pseudomonadota phylum is known to contain several pathogens and putative pathobionts (58) and Actinomycetota, Bacteriodota, and Fusobacteriota contribute to various physiological functions, including digestion and immune modulation (59). In early lactation, milk iron, manganese, copper, and selenium were correlated with the differentially abundant taxa while in established lactation, milk iron, manganese, selenium, calcium, and sodium were correlated with milk bacteria. Only iron, manganese and selenium were associated with milk bacteria at both stages of lactation, and iron and manganese had the greatest number of associations with differentially abundant ESVs. These minerals were correlated with members of the phyla Pseudomonadota (Fe and Mn) in early lactation, with Actinomycetota (Fe and Mn) in established lactation and with Bacillota in both stages of lactation (Mn and Se).

### Associations of maternal diet with milk mineral concentrations

Previous research had focused on the maternal intake of fatty acids and vitamins and their association with human milk composition (6, 8), but evidence regarding milk minerals is limited and generally considered less related to maternal diet (6, 60). Previous studies had reported correlations between maternal intake and milk concentrations for selenium (7), zinc (61), calcium (62), and iron (3, 62). In contrast, other studies had not observed significant associations between maternal mineral intakes and milk concentrations for zinc (3, 63), calcium (3), and potassium (3, 62), which aligns with our data. In our study, the observation that all significant correlations between maternal mineral intakes and milk mineral concentrations were negative and weak is intriguing. Other studies in low-socioeconomic communities have also observed inverse associations between milk concentrations and maternal diet for zinc (12, 62, 64, 65) suggesting that during maternal deficiencies there is mobilization and transport of the mineral to the milk (62, 66, 67). Importantly, mothers in our study had inadequate intakes of several minerals based on dietary INCAP requirements. With regards to INCAP’s suggested milk mineral concentrations, there was evidence of both inadequate and adequate milk concentrations in mothers’ milk during both early and established lactation, suggesting weak correlations between intakes and milk concentration. Our correlation circle and heat maps further supported our observation that milk mineral concentrations underscored changes in the milk microbiome in contrast to maternal dietary intakes in mothers experiencing multiple micronutrient deficiencies during both early and established lactation.

Others have suggested that bioavailability of minerals from food sources may contribute to the negative and weak correlations between maternal mineral intake and milk mineral concentrations (68). The *Mam-*Mayan community follows the “three sisters” diet, a diet based on corn, squash and beans, where most of their food sources are plant sources rather than animal sources and is based on starchy staples (mostly maize), legumes where only one third consumed flesh foods (mostly chicken or beef), and dark green leafy vegetables (30). In our analysis, we did not consider the source of the maternal mineral intake (i.e., from animal or plant sources), which can affect the bioavailability of minerals. Therefore, the limited mineral bioavailability, due to plant foods as the main source of nutrients and the demonstrated inadequate intakes for multiple minerals, may be linked to widespread low availability and inadequate intakes of minerals and trace elements.

The weak correlations of maternal sodium and potassium at early lactation, and maternal copper and selenium at established lactation with milk mineral concentrations observed in all mothers could be associated with homeostatic processes, such as active transport mechanisms in the mammary gland. These have been described for iron and copper (12, 60), suggesting that regardless of the maternal mineral intake, the milk mineral concentrations remain independent from the maternal diet due to these homeostatic processes. Other factors could influence more milk mineral concentrations than maternal diet, such as lactation stage, maternal and infant age, parity, maternal physiological status (e.g., bone metabolism, renal function, hormonal regulation), maternal chronic diseases (e.g., diabetes, thyroid disorders), infections, geographic location (e.g., soil composition) and maternal genetic variants (7, 13).

### Associations of maternal diet with milk microbiome composition

As for maternal diet and milk microbiome, some associations have been reported with macronutrients, fiber, poly- and mono-unsaturated fatty acids and vitamins (27, 69–74). Yet, our results show no direct association between maternal mineral intake and milk microbiome; only dietary selenium was positively correlated with *E. americana,* and dietary zinc was positively correlated with *S. hominis*. Moreover, it is possible that statements about a strong link of maternal diet with milk microbiome might apply to other nutrients, but not minerals.

We found that the mineral intake below the reference values for iron, manganese, calcium, selenium, and potassium in our population highlighted a milk ecosystem with multiple co-existing deficiencies in minerals and trace elements. Previous studies reporting associations between maternal diet and milk microbiome had not described dietary adequacy (71–73); with the exception of one study, which focused on fatty acids and vitamins in well-nourished lactating women (74). Further research is needed regarding the differences in milk microbiota and micronutrient intakes in communities already experiencing multiple micronutrient deficiencies.

### Associations of milk mineral concentrations with milk microbiome

Milk mineral concentrations and milk microbiome composition are dynamic and vary throughout lactation with the nutritional composition of human milk linked to the composition of the milk microbiota (21, 36). Only one study had analyzed the association between milk minerals and milk bacteria, but had not considered the difference between early and established lactation (21). Sanjulián et al. observed that calcium was negatively correlated with *Streptococcus, Prevotella,* Actinomycetota, and Bacteroidota; magnesium positively correlated with *Streptococcus* abundance; and selenium negatively correlated with *Staphylococcus.* In contrast, we observed a positive correlation of calcium with *Amaricoccus* spp. and *Gardnerella* spp. at established lactation, no significant correlations with magnesium, and selenium was positively correlated with *Staphylococcus caprae* at early lactation and with *Turicibacter bilis* at established lactation. In the gut microbiome, it has been emphasized that levels of mineral intake must be tightly regulated to prevent colonization of gut pathogens while maintaining a healthy immune response to invading bacteria (17). This tight regulation may also be relevant in the mammary gland to prevent the colonization of microbial pathogens in the milk.

### Individual minerals and trace elements associated with the milk microbiome

In our study we uncovered three minerals associated with the milk microbiome in both early and established lactation: iron, manganese, and selenium. We further detail these associations below, as iron and manganese have been associated with nutritional immunity (15–17, 75, 76). Nutritional immunity is a process by which the host reduces mineral availability to the bacteria, intended to inhibit bacterial growth (17). This process has been reported in the gut for iron, manganese, zinc and copper (15–17, 75, 76).

#### Iron

In the human body, iron is essential for oxygen transport, DNA metabolism, and mitochondrial function (77). For bacteria, iron is also a co-factor in iron-containing proteins in redox reactions, metabolic pathways, and electron transport chain mechanisms (77). In early lactation, iron was correlated with species from the Pseudomonadota phylum (in particular *Pseudomonas*), while in established lactation it was correlated with Bacillota and Actinomycetota. Previous studies have not associated milk iron with *Pseudomonas* but there is evidence of iron affinity in some *Pseudomonas* species, such as the opportunistic species *P. aeruginosa* that has a high capacity to acquire iron and use it as a virulence determinant (78, 79). This iron affinity might be shared with other taxa in the Pseudomonadota phylum, which could explain its several associations with iron in this study. Other potential pathogens like *E. coli, Salmonella,* and *Shigella* are also siderophilic species, while beneficial bacteria such as *Bifidobacteria* or *Lactobacillus* have low iron requirements (80). Our analyses support negative correlations between *Bifidobacteria* and *Lactobacilli* and milk iron concentrations, and the slightly higher mean concentration of milk iron could explain the low abundance of these bacterial taxa in our milk samples (81, 82). Importantly, Pseudomonadota are the first gut colonizers, helping the gut reach a full anaerobic status (83, 84). Thus, iron in human milk may play a role in facilitating gut early colonization by Pseudomonadota, and may have implications for infant growth and nutritional status through alterations in the gut microbiome (80).

#### Manganese

In our study, manganese was correlated with species of the Bacillota (*Staphylococcus hominis* and *Staphylococcus* spp.) and Pseudomonadota (*Ewingella americana*) phyla in early lactation, and in established lactation it continued to be correlated with Bacillota (*Streptococcus, Planococcus chinensis* and *Turicibacter bilis*) but also with Bacteroidota (*Prevotella* spp. and *Prevotella jejuni*) and Actinomycetota (*Luteococcus peritonei* and *Pseudarthrobacter polychromogenes*). Manganese is an essential nutrient for intracellular activities in the human; it functions as a cofactor for a variety of enzymes involved in development, digestion, reproduction, antioxidant defense, energy production, immune response and regulation of neuronal activities (85). However, at elevated levels, manganese can have neurotoxic effects (86). Evidence on its association with bacteria is scarce but, in the gut, manganese has been associated with Bacillota and Bacteroidota (87), which aligns with our findings. Manganese has also been associated with immune function, bacterial survival, and a capacity to promote virulence of pathogens (16, 88). In the gut, deficiencies in dietary manganese have been linked to increased risk of infection by *Staphylococcus aureus* (17). In cows, it has been observed that manganese is needed by *S. uberis* (89) and *Lactobacillus rhamnosus* (90) to grow. Manganese seems to stop acidification within 1 day of nongrowing cells by affecting the lactococcal acidification rate (90). Similarly, also in cows, there is evidence that the supplementation of manganese decreased the relative abundance of *Treponema* within phylum *Spirochaetes* (91). Although none of these species were observed in our results, the evidence in cow’s milk shows that manganese interacts with bacteria and it is necessary for the growth of certain species, which might occur in human milk. However further research is needed to understand their specific role in milk microbiome and analyze if the observations in the gut microbiome and in animals are similar in human milk.

#### Iron and manganese

We report that milk iron and manganese together made 64 and 75% of the correlations with bacterial community structure at early and established lactation, respectively, although these minerals were not reported in previous studies. Interestingly, in the gut, iron and manganese can influence each other’s absorption as both metals use the same transporters (88, 92, 93). For example, in the presence of *Mycobacterium tuberculosis* and *Salmonella enterica,* the host reduces mineral availability by redirecting storage of cellular iron, manganese, and magnesium from the phagolysosome to the cytoplasm. Although not investigated in milk, this nutritional immunity could be operational in milk specifically with iron and manganese, as they are the minerals with the most associations with bacteria at early and established lactation.

#### Selenium

Previous research in milk microbiome reported that selenium was negatively correlated with *Staphylococcus* (21), which partially aligns with our findings, as selenium correlated positively with *S. caprae*. A recent study in rats deficient in selenium reported higher levels of Bacillota, although at genus level they did not identify *Staphylococcus* (94). A similar association could be happening in our samples, as selenium was below the INCAP reference values at both stages of lactation and it was positively correlated with *S. caprae* at early lactation and with *Turicibacter bilis* at established lactation, both members of the Bacillota phylum. There is evidence in this same population that SCM is associated with higher milk selenium (38). Selenium was one of the minerals that showed associations with milk microbiome in both stages of lactation so possibly SCM is a factor interfering in this association and further research that evaluates the association between milk selenium and milk bacteria in SCM and non-SCM mothers is needed. Selenium is an essential micronutrient for humans, as it is a component of selenoproteins. Selenoproteins are important for thyroid hormone metabolism, several immune system antioxidant defense processes (e.g., glutathione peroxidase), and for the intracellular control of oxidation and reduction reactions (86). Importantly, an excessive exposure to selenium can have adverse effects on health (86). It has been further reported that selenium has inhibitory effects on Staphylococci growth, as the supplementation of selenium inhibits the growth of *S. aureus* in bovine milk. As selenium is essential for the normal function of the immune system, it has also been suggested that mothers with higher levels of milk selenium could have an immune system more capable of reducing levels of *Staphylococcus* (21). Similarly, there is evidence that selenium supplementation increases the diversity and richness of gut microbiota (95), including health-relevant taxa, which highlights its importance in milk microbiome. Of note, it has been reported that ¼ of all bacteria have selenoproteins, so they require selenium for their growth and metabolism (96). This could explain why this was one of the minerals that mainly correlated with milk bacteria at both stages of lactation. In fact, a study evaluated *in vivo* the nutritional availability of selenometabolites and concluded that gut microbiota might contribute to the selenium nutritional availability (97), which again might also happen in the milk microbiome.

We recognize this study had certain limitations. A potential weakness of the dataset was the necessary inclusion of mothers with subclinical mastitis (SCM) to increase sample size, as the presence of SCM can affect the milk mineral concentrations (38). Our mothers were not supplemented with any micronutrients, but they consumed fortified foods which could modify the milk mineral concentrations. We acknowledge the value of more advanced models for future research, that should monitor larger cohorts of lactating mothers over time and include more detailed dietary intake data, to explore the independent contribution of each variable to milk mineral concentration and milk microbial diversity using more advanced statistical modeling. The transferability of our results is limited due to the unique characteristics of the *Mam*-Mayan population, including their traditional diet, environmental exposures, and cultural practices, previously described in detail (30, 55, 98). However, our findings may still be valuable to other indigenous communities with similar dietary patterns, lifestyle factors, and socioeconomic conditions. Additionally, populations experiencing nutrient deficiencies or residing in low- and middle-income countries (LMICs) with comparable nutritional and environmental challenges may also benefit from these insights. Future research involving diverse populations could help determine the extent to which these associations hold across different settings.

## Conclusion

In summary, our findings reveal a complex and dynamic interplay between milk minerals and the milk microbiome at early and established lactation. Results revealed a milk ecosystem where concentrations in milk minerals and trace elements were differentially associated with the milk microbiome. Only milk iron, manganese, and selenium were associated with milk bacteria in both stages of lactation including members of the phyla Pseudomonadota with Fe and Mn in early lactation, Actinomycetota with Fe and Mn in established lactation, and Bacillota in both stages of lactation with Mn and Se. These bidirectional microbial correlations with milk minerals revealed that certain bacteria may thrive or be inhibited depending on the presence of specific minerals during early and established lactation.

Subsequent research will be required to better understand causation and the mechanisms by which milk minerals and milk microbiome interact and if these interactions are the same in nutrient-deficient and nutrient-adequate mothers. These future investigations should include milk volume in their analyses, distinguish between the food sources (animal or plant foods), and consider the timing between maternal meals and the milk sample collection. Additionally, future studies should explore if these modifications between milk minerals, trace elements and the milk microbiome might be linked to the high rates of growth faltering in breastfed infants in Guatemala and to impaired growth in other LMIC countries.

## Data Availability

The datasets presented in this study can be found in online repositories at: https://www.ncbi.nlm.nih.gov/bioproject/PRJNA1258222.
