# Supplementary material for: Milk mineral composition is strongly associated with the human milk microbiome
Source: Front Nutr. 2025 May 21;12:1550292. doi: 10.3389/fnut.2025.1550292 (PMC12133538; doi:10.3389/fnut.2025.1550292)

**Supplementary Material**

## **SUPPLEMENTARY TABLES**

| **Supplementary Table 1.  Population Characteristics, Maternal Nutrient Intake and Milk Mineral Concentrations** | | | | |
| --- | --- | --- | --- | --- |
|  | **Total  N = 77** | **Early Stage ^1^ n = 38** | **Established Stage ^2^ n = 39** | **p-value** |
| **INFANT CHRACTERISTICS** | | | | |
| Age, days | 83 ± 66 | 18 ± 9 | 147 ± 17 | <0.001*** |
| **Sex, n (%)** |  |  |  | 0.3 |
| Male, | 43 (55.84) | 21 (55.26) | 22 (56.41) |  |
| Female | 34 (34.69) | 17 (44.74) | 17 (43.59) |  |
| **MATERNAL CHARACTERISTICS** | | | | |
| **Age, y (%)** |  |  |  |  |
| <19 | 17 ± 1 (27.3) | 17 ± 2 (21.1) | 17 ± 1 (33.3) | 0.638 |
| ≥19 | 27 ± 6 (72.7) | 27 ± 6 (78.9) | 28 ± 6 (66.7) | 0.523 |
| Average (± stdev) | 24 ± 7 | 25 ± 7 | 24 ± 7 | 0.724 |
| Height, cm | 146.6 ± 5.5 | 148.2 ± 5.3 | 145.1 ± 5.3 | 0.014* |
| Weight, kg | 50.6 ± 8 | 51.2 ± 7.6 | 49.9 ± 8.4 | 0.521 |
| **BMI, kg/m^2^ (% of mothers)** |  |  |  |  |
| Underweight (BMI:<18.5) | 17.0 ± 0.7 (3.9) | 17.4 (2.6) | 16.8 ± 0.9 (5.1) | 0.667 |
| Normal (BMI:18.5 – 24.9) | 22.4 ± 3.3 (70.1) | 22 ± 3.1 (71.1) | 22.8 ± 3.5 (69.2) | 0.096 |
| Overweight, (BMI: 25 – 29.9) | 26.6 ± 3.3 (23.4) | 26.6 ± 3.1 (23.7) | 26.6 ± 3.5 (23.1) | 1.0 |
| Obesity, (BMI: >30) | 35.1 ± 2.7 (2.6) | 33.2 (2.6) | 37.0 (2.6) | - |
| Average (± stdev) | 23.5 ± 3.3 | 23.3 ± 3.1 | 23.8 ± 3.5 | 0.541 |
| **Parity** |  |  |  | 0.108 |
| Primiparous, n (%) | 31 (40.8) | 15 (39.5) | 16 (42.1) |  |
| Multiparous, n (%) | 45 (59.2) | 23 (60.5) | 22 (57.9) |  |
| **MATERNAL NUTRIENT INTAKE ^3^** | | | | |
| Water, g | 1435 ± 483 | 1604 ± 398^a^ | 1270 ± 506 ^b^ | 0.002** |
| Energy, kcal | 1292.9 ± 373.2 | 1422.7 ± 319.9 ^a^ | 1166.4 ± 381.5 ^b^ | 0.002** |
| Protein, g | 39.9 ± 17.5 | 45.9 ± 18.2 ^a^ | 34 ± 14.8 ^b^ | 0.002** |
| Lipids, g | 19.7 ± 7.9 | 22.5 ± 7.1 ^a^ | 16.9 ± 7.7 ^b^ | 0.002** |
| Saturated fats, g | 3.6 ± 1.9 | 4.3 ± 2 ^a^ | 2.9 ± 1.6 ^b^ | 0.001*** |
| Monounsaturated fats, g | 5.8 ± 2.8 | 6.8 ± 2.6 ^a^ | 4.8 ± 2.6 ^b^ | <0.001*** |
| Polyunsaturated fats, g | 7.5 ± 2.8 | 8.1 ± 2.1 ^a^ | 6.9 ± 3.3 ^b^ | 0.047* |
| Cholesterol, mg | 72.1 ± 93.8 | 97.9 ± 107.2 ^a^ | 47 ± 71.4 ^b^ | 0.017* |
| Carbohydrates, g | 257.9 ± 71.7 | 279.5 ± 63.4 ^a^ | 236.9 ± 73.9 ^b^ | 0.008* |
| Sugars, g | 59.5 ± 23.4 | 67.8 ± 24.3 ^a^ | 51.4 ± 19.6 ^b^ | 0.002* |
| Fiber, g | 27.3 ± 9.3 | 28.5 ± 7 | 26.2 ± 11.0 | 0.283 |
| Calcium, mg | 682.3 ± 193.5 | 728.6 ± 165.2 ^a^ | 637.1 ± 210 ^b^ | 0.037* |
| Copper, µg | 1238 ± 437.3 | 1325 ± 372.5 | 1153 ± 482.6 | 0.085 |
| Iron, µg | 12435.6 ± 13141.3 | 14748 ± 18002 | 10182 ± 4391 | 0.128 |
| Magnesium, mg | 412.4 ± 125.9 | 433.1 ± 104.7 | 392.3 ± 142 | 0.156 |
| Manganese, mg | 1.4 ± 0.6 | 1.5 ± 0.52 | 1.3 ± 0.7 | 0.068 |
| Potassium, mg | 1704.1 ± 640.2 | 1766.2 ± 457.9 | 1643.5 ± 779.7 | 0.401 |
| Selenium, µg | 49.7 ± 21.5 | 57.8 ± 21.8 ^a^ | 41.8 ± 18.3 ^b^ | <0.001*** |
| Sodium, mg | 2561.2 ± 2053.7 | 3422.1 ± 2510.8 ^a^ | 1722.4 ± 913 ^b^ | <0.001*** |
| Zinc, µg | 7714.2 ± 4299.2 | 8781 ± 5311 ^b^ | 66676 ± 2694 ^a^ | 0.031* |
| **MILK MINERAL CONCENTRATION ^3^** | | | | |
| Calcium, mg/L | 261.1 ± 42.5 | 273.2 ± 47 ^a^ | 249.4 ± 34.3 ^b^ | 0.013* |
| Copper, µg/L | 389.8 ± 158.6 | 509 ± 108.7 ^a^ | 273.6 ± 103.5 ^b^ | <0.001* |
| Iron, µg/L | 381.2 ± 254.8 | 393.5 ± 193.2 ^a^ | 369.1 ± 305.4 ^b^ | 0.678 |
| Magnesium, mg/L | 29 ± 7.9 | 22.7 ± 4.3 ^b^ | 35.1 ± 5.4 ^a^ | <0.001*** |
| Manganese, µg/L | 9.4 ± 6.6 | 8.8 ± 5.1 ^b^ | 9.9 ± 7.8 ^a^ | 0.443 |
| Potassium, mg/L | 498.3 ± 88.2 | 551.2 ± 80 ^a^ | 446.8 ± 61.2 ^b^ | <0.001*** |
| Selenium, µg/L | 13.6 ± 4.4 | 15.6 ± 3.9 ^a^ | 11.7 ± 4 ^b^ | <0.001*** |
| Sodium, mg/L | 153 ± 125.4 | 188.5 ± 124.8 ^a^ | 118.4 ± 117.3 ^b^ | 0.013* |
| Zinc, µg/L | 2707.3 ± 1989.9 | 4035.5 ± 2004.1 ^a^ | 1395.7 ± 656.6 ^b^ | <0.001*** |

Note: ^1^ Early stage is defined from 5 to 46 days postpartum; ^2^ Late stage is defined from 109 – 184 days postpartum. ^3^ Values are arithmetic means ± SDs. Means labelled with an “a” are higher than means labelled with a “b” among the values in the same row.

**Supplementary Figure 1 and 2 –** Heatmap of a univariate Spearman correlation matrix between milk mineral concentrations and the maternal mineral intake at (A) early and (B)

established lactation. Red squares represent positive correlations, and blue squares represent negative correlations. The intensity of the colors represents the degree of association between the

milk mineral concentrations and the maternal diet as measured by Spearman’s correlations. The stars represent significant correlations (FDR < 0.1).

**Supplementary Figure 1 - Early lactation**


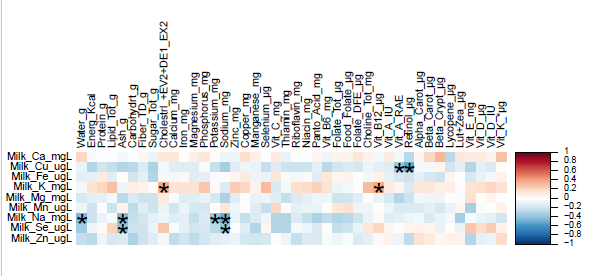


**Supplementary Figure 2 - Established lactation**


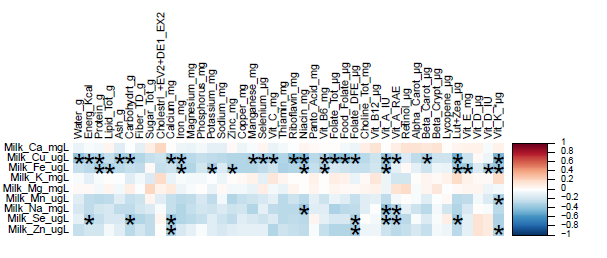


**Supplementary Figure 3 -** Beta-diversity analyses segregated both lactation stages, along the Canonical Correspondence Analysis (CCA) (*p* = <0.001), Redundancy Analysis (RDA) (*p* < 0.001) and Principal Coordinate Analysis (PCoA) (*p* = <0.001) ordinations


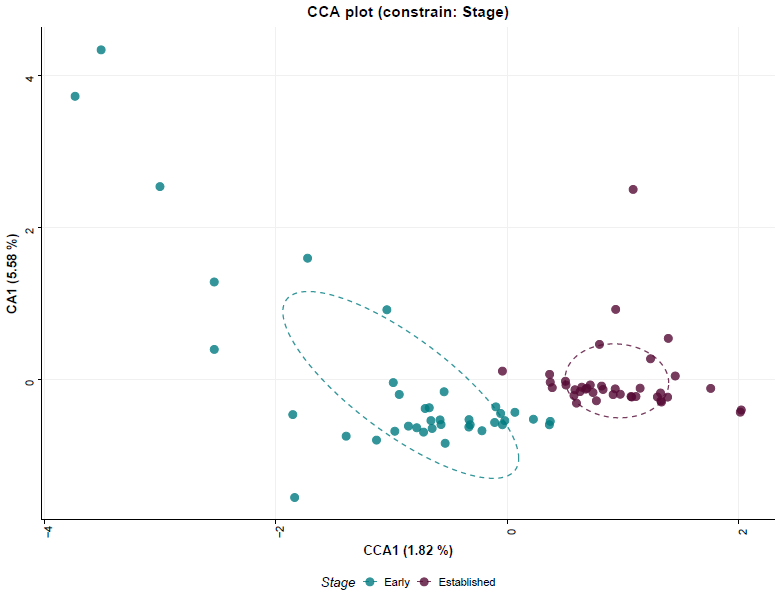


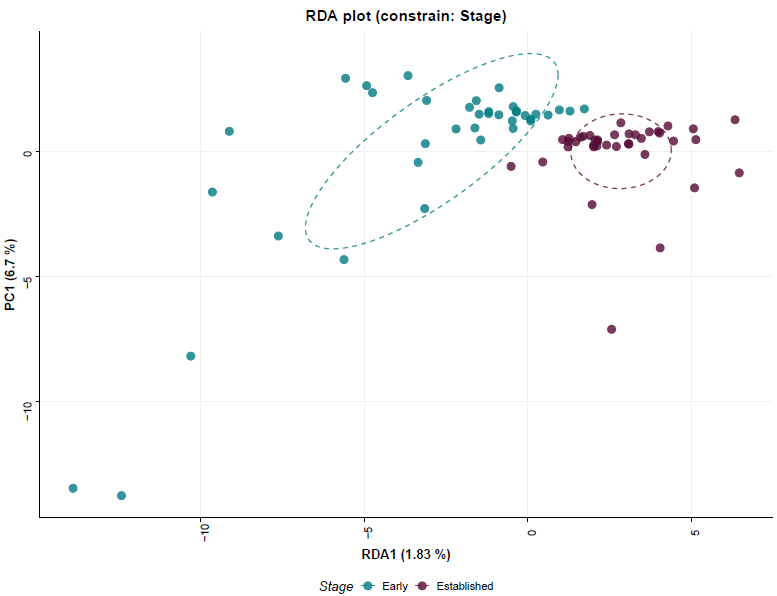


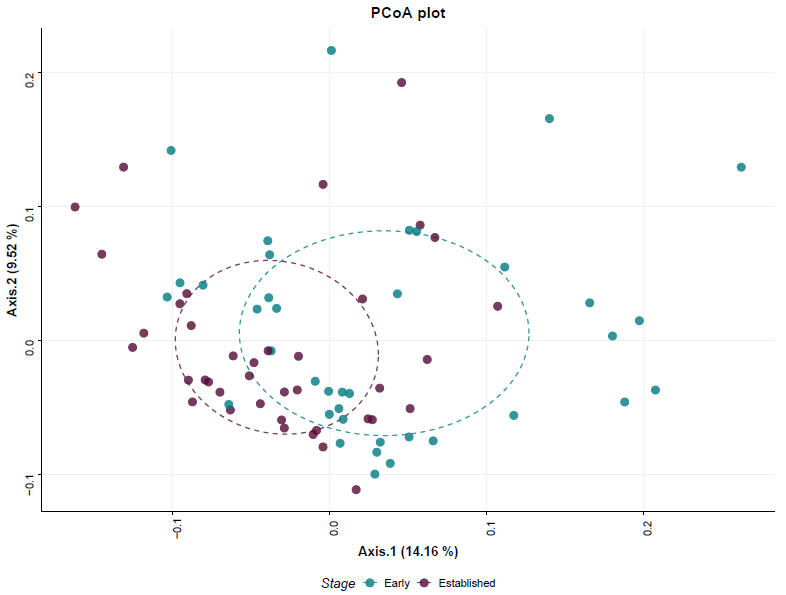

Supplement: Supplementary file 1 [file Table_1.docx]
